# Postoperative Outcomes in Normotensive and Hypertensive Pheochromocytomas: An International Study

**DOI:** 10.1210/clinem/dgaf154

**Published:** 2025-03-06

**Authors:** Marta Araujo-Castro, Aura Herrera, Yanbo Wang, Zhicheng Wang, Maciej Śledziński, Andrzej Hellmann, Marco Raffaelli, Francesco Pennestrì, Mark Sywak, Alexander J Papachristos, Fausto F Palazzo, Tae-Yon Sung, Byung-Chang Kim, Yu-mi Lee, Fiona Eatock, Hannah Anderson, Maurizio Iacobone, Albertas Daukša, Ozer Makay, Yigit Turk, Hafize Basut Atalay, Els J M Nieveen van Dijkum, Anton F Engelsman, Isabelle Holscher, Gabriele Materazzi, Leonardo Rossi, Chiara Becucci, Susannah L Shore, Clare Fung, Alison Waghorn, Radu Mihai, Sabapathy P Balasubramanian, Arslan Pannu, Shuichi Tatarano, David Velázquez-Fernández, Julie A Miller, Hazel Serrao-Brown, Yufei Chen, Marco Stefano Demarchi, Reza Djafarrian, Helen Doran, Kelvin Wang, Michael J Stechman, Helen Perry, Johnathan Hubbard, Cristina Lamas, Philippa Mercer, Janet MacPherson, Supanut Lumbiganon, María Calatayud, Felicia Alexandra Hanzu, Oscar Vidal, Cesar Minguez Ojeda, Theodosios Papavramidis, Pablo Rodríguez de Vera Gómez, Abdulaziz Aldrees, Tariq Altwjry, Nuria Valdés, Cristina Álvarez-Escola, Iñigo García Sanz, Concepción Blanco Carrera, Laura Manjón-Miguélez, Paz De Miguel Novoa, Mónica Recasens, Rogelio García Centeno, Cristina Robles Lázaro, Klaas Van Den Heede, Sam Van Slycke, Theodora Michalopoulou, Sebastian Aspinall, Ross Melvin, Joel Wen Liang Lau, Wei Keat Cheah, Man Hon Tang, Han Boon Oh, John Ayuk, Kevin Verhoeff, Robert P Sutcliffe, Alessandro Parente

**Affiliations:** Department of Endocrinology, Hospital Universitario Ramón y Cajal, Madrid 28034, Spain; Instituto de Investigación Ramón y Cajal (YRICIS), Madrid 28034, Spain; Department of Endocrinology and Nutrition, University Hospital Reina Sofia, Cordoba 14004, Spain; Department of Urology, The First Affiliated Hospital of Jilin University, Changchun, Jilin 2699, China; Department of Urology, The First Affiliated Hospital of Jilin University, Changchun, Jilin 2699, China; Division of General, Endocrine and Transplant Surgery, Medical University of Gdańsk, Gdańsk 80210, Poland; Division of General, Endocrine and Transplant Surgery, Medical University of Gdańsk, Gdańsk 80210, Poland; UOC Chirurgia Endocrina e Metabolica, Fondazione Policlinico Universitario Agostino Gemelli IRCCS, Centro di Ricerca in Chirurgia Endocrina e dell'Obesità, Università Cattolica del Sacro Cuore, Rome 00136, Italy; UOC Chirurgia Endocrina e Metabolica, Fondazione Policlinico Universitario Agostino Gemelli IRCCS, Centro di Ricerca in Chirurgia Endocrina e dell'Obesità, Università Cattolica del Sacro Cuore, Rome 00136, Italy; Endocrine Surgical Unit, Royal North Shore Hospital, Northern Sydney Local Health District, Sydney, NSW 2065, Australia; Endocrine Surgical Unit, Royal North Shore Hospital, Northern Sydney Local Health District, Sydney, NSW 2065, Australia; Department of Endocrine Surgery, Hammersmith Hospital, London W12 OHS, UK; Department of Surgery, Asan Medical Center, University of Ulsan College of Medicine, Seoul 05505, Republic of Korea; Department of Surgery, Asan Medical Center, University of Ulsan College of Medicine, Seoul 05505, Republic of Korea; Department of Surgery, Asan Medical Center, University of Ulsan College of Medicine, Seoul 05505, Republic of Korea; Department of Endocrine Surgery, Royal Victoria Hospital, Belfast BT12 6BA, UK; Department of Endocrine Surgery, Royal Victoria Hospital, Belfast BT12 6BA, UK; Endocrine Surgery Unit, Department of Surgery, Oncology and Gastroenterology, University of Padua, Padua 35143, Italy; Department of Surgery, Lithuanian University of Health Sciences, Kaunas 44307, Lithuania; Ozel Saglik Hospital, Centre of Endocrine Surgery, Izmir 35000, Turkey; Division of Endocrine Surgery, General Surgery Department, Ege University Hospital, Izmir 35100, Turkey; Ozel Saglik Hospital, Centre of Endocrine Surgery, Izmir 35000, Turkey; Department of Surgery, Amsterdam UMC, University of Amsterdam, Cancer Center Amsterdam, Amsterdam 1081, the Netherlands; Department of Surgery, Amsterdam UMC, University of Amsterdam, Cancer Center Amsterdam, Amsterdam 1081, the Netherlands; Department of Surgery, Amsterdam UMC, University of Amsterdam, Cancer Center Amsterdam, Amsterdam 1081, the Netherlands; Endocrine Surgery Unit, University Hospital of Pisa, Pisa 56100, Italy; Endocrine Surgery Unit, University Hospital of Pisa, Pisa 56100, Italy; Endocrine Surgery Unit, University Hospital of Pisa, Pisa 56100, Italy; Department of Endocrine and Breast Surgery, Royal Liverpool and Broadgreen University Hospitals Trust, Liverpool L7 8XP, UK; Department of Endocrine and Breast Surgery, Royal Liverpool and Broadgreen University Hospitals Trust, Liverpool L7 8XP, UK; Department of Endocrine and Breast Surgery, Royal Liverpool and Broadgreen University Hospitals Trust, Liverpool L7 8XP, UK; Department of Endocrine Surgery, Churchill Cancer Centre, Oxford University Hospitals NHS Foundation Trust, Oxford OX3 9DU, UK; Department of General Surgery, Sheffield Teaching Hospitals Foundation Trust, Sheffield S10 2JF, UK; Department of General Surgery, Sheffield Teaching Hospitals Foundation Trust, Sheffield S10 2JF, UK; Department of Urology, Graduate School of Medical and Dental Sciences, Kagoshima University, Kagoshima, Japan; Servicio de Cirugía Endocrina y Laparoscopia Avanzada, Departamento de Cirugía, Instituto Nacional de Ciencias Médicas y Nutrición Salvador Zubirán, Mexico City 14080, Mexico; Endocrine Surgery Unit, The Royal Melbourne Hospital, Melbourne, VIC 3050, Australia; Endocrine Surgical Unit, Royal North Shore Hospital, Northern Sydney Local Health District, Sydney, NSW 2065, Australia; Cedars-Sinai Medical Center, Department of Surgery, Los Angeles, CA 90048, USA; Department of Thoracic and Endocrine Surgery and Faculty of Medicine, University Hospitals of Geneva, Geneva 1211, Switzerland; Department of Thoracic and Endocrine Surgery and Faculty of Medicine, University Hospitals of Geneva, Geneva 1211, Switzerland; Department of Endocrine Surgery, Salford Royal Hospital, Salford M6 8HD, UK; Department of Endocrine Surgery, Salford Royal Hospital, Salford M6 8HD, UK; Department of Endocrine Surgery, University Hospital Wales, Cardiff CF14 4XW, UK; Department of Endocrine Surgery, University Hospital Wales, Cardiff CF14 4XW, UK; Department of Endocrine Surgery, St Thomas’ Hospital, London SE1 7EH, UK; Endocrinology & Nutrition Department, Hospital Universitario de Albacete, Albacete 02008, Spain; Endocrine Surgical Unit, Department of General Surgery, Christchurch Hospital, Christchurch 4710, New Zealand; Department of Anaesthesia, Christchurch Hospital, Christchurch 4710, New Zealand; Department of Surgery, Division of Urology, Khon Kaen University, Khon Kaen 40002, Thailand; Endocrinology & Nutrition Department, Hospital Universitario 12 de Octubre, Madrid 28041, Spain; Endocrinology & Nutrition Department, Hospital Clinic University Barcelona, Barcelona 08036, Spain; Endocrine Surgery Department, Hospital Clinic University Barcelona, Barcelona 08036, Spain; Urology Department, Hospital Universitario Ramón y Cajal, Madrid 28034, Spain; 1st Propedeutic Department of Surgery, AHEPA University Hospital, Aristotle University of Thessaloniki, Thessaloniki 546 36, Greece; Endocrinology & Nutrition Department, Hospital Universitario Virgen de la Macarena, Sevilla 41009, Spain; Endocrine Surgery, Department of General Surgery, King Abdulaziz Medical City, Riyadh 14611, Saudi Arabia; Endocrine Surgery, Department of General Surgery, King Abdulaziz Medical City, Riyadh 14611, Saudi Arabia; Endocrinology & Nutrition Department, Hospital Universitario Cruces Barakaldo, Vizcaya 856350, Spain; Endocrinology & Nutrition Department, Hospital Universitario La Paz, Madrid 28046, Spain; General & Digestive Surgery Department, Hospital Universitario de La Princesa, Madrid 28006, Spain; Endocrinology & Nutrition Department, Hospital Universitario Príncipe de Asturias, Madrid 28805, Spain; Endocrinology & Nutrition Department, Hospital Universitario Central de Asturias, Oviedo 33011, Spain; Instituto de Investigación Sanitaria del Principado de Asturias (ISPA), Oviedo 33011, Spain; Endocrinology & Nutrition Department, Hospital Clínico San Carlos, Madrid 28040, Spain; Endocrinology & Nutrition Department, Institut Català de la Salut Girona, Girona 17005, Spain; Endocrinology & Nutrition Department, Hospital Universitario Gregorio Marañón, Madrid 28007, Spain; Endocrinology & Nutrition Department, Hospital Universitario de Salamanca, Salamanca 37007, Spain; General and Endocrine Surgery, Onze-Lieve-Vrouw (OLV) Hospital Aalst-Asse-Ninove, Aalst, Belgium; General and Endocrine Surgery, Onze-Lieve-Vrouw (OLV) Hospital Aalst-Asse-Ninove, Aalst, Belgium; Department of Endocrinology and Nutrition, Joan XXIII University Hospital, Tarragona 43005, Spain; Department of General Surgery, Aberdeen Royal Infirmary, Aberdeen AB25 2ZN, UK; Department of General Surgery, Aberdeen Royal Infirmary, Aberdeen AB25 2ZN, UK; Division of Breast & Endocrine Surgery, Department of General Surgery, Ng Teng Fong General Hospital (NTFGH), National University Health System (NUHS), Singapore 609606, Singapore; Division of Breast & Endocrine Surgery, Department of General Surgery, Ng Teng Fong General Hospital (NTFGH), National University Health System (NUHS), Singapore 609606, Singapore; Division of Breast & Endocrine Surgery, Department of General Surgery, Ng Teng Fong General Hospital (NTFGH), National University Health System (NUHS), Singapore 609606, Singapore; Division of Breast & Endocrine Surgery, Department of General Surgery, Ng Teng Fong General Hospital (NTFGH), National University Health System (NUHS), Singapore 609606, Singapore; Department of Endocrinology, Queen Elizabeth Hospital B15 2GW, Birmingham, UK; Department of Surgery, Division of General Surgery, University of Alberta, Faculty of Medicine & Dentistry, Edmonton, AB, Canada T6G 2R3; Department of Hepatopancreatobiliary and Liver Transplant Surgery, Queen Elizabeth Hospital, Birmingham B15 2GW, UK; Department of Hepatopancreatobiliary and Liver Transplant Surgery, Queen Elizabeth Hospital, Birmingham B15 2GW, UK; Institute of Liver Studies, King's College Hospital NHS Foundation Trust, Denmark Hill, London SE5 9RS, UK

**Keywords:** pheochromocytoma, normotensive, hypertensive, Clavin-Dindo, surgical outcomes

## Abstract

**Context:**

Postoperative outcomes of patients with normotensive pheochromocytomas are poorly documented.

**Objective:**

We aimed to evaluate the impact of preoperative hypertension on postoperative outcomes following adrenalectomy for pheochromocytoma.

**Methods:**

An international retrospective study of patients undergoing adrenalectomy for pheochromocytoma in 46 centers between 2012 and 2022 was performed. Hypertensive and normotensive pheochromocytoma were defined respectively by the presence or absence of hypertension history before or at the time of pheochromocytoma diagnosis. To evaluate differences in postoperative outcomes between hypertensive and normotensive patients, propensity score matched (PSM) analysis was performed.

**Results:**

Among 2016 patients with pheochromocytoma, 1034 (51.2%) had preoperative hypertension and 982 (49.8%) were normotensive. Hypertensive patients were 4.5 years older (*P* < .001), had a higher prevalence of type 2 diabetes (*P* < .001), had a higher median Charlson Comorbidity Index (2.0 vs 1.0; *P* < .001), and had an American Society of Anesthesiologists score of III to IV more frequently (41% vs 19.9%; *P* < .001) than normotensive patients. Nonadjusted analysis demonstrated that hypertensive patients had longer operative time (115.0 vs 103.5 minutes; *P* = .026), higher rate of vasopressors at skin closure (19.7% vs 15.4%; *P* = .013), more perioperative blood transfusions (7.7% vs 5.0%; *P* = .016), and an increased complication rate (21.6% vs 17.7%; *P* = .029). However, after 1:1 PSM, we found that readmission, complications, and serious complications were similar between cohorts.

**Conclusion:**

Patients with hypertensive pheochromocytomas have a higher risk of postoperative complications than normotensive patients due to the association of hypertension with a higher burden of comorbidities and older age. However, hypertension is not an independent risk factor of postoperative complications after pheochromocytoma surgery.

Pheochromocytomas are rare neuroendocrine tumors of the adrenal medulla that produce and release catecholamines (epinephrine, norepinephrine, and dopamine) and their metabolites (1, 2). Their clinical presentation is highly variable and depends on multiple factors, including tumor size, extent of locoregional or distant involvement, catecholamine secretion and its hereditary or sporadic nature, among others. Despite this, hypertension is considered the most frequent symptom in these patients (3). The prevalence of hypertension at the time of the pheochromocytoma diagnosis is quite variable, ranging from 50% to 95% depending on the definition of hypertension, the study population, and other factors (4, 5). Thus, it means that up to 50% of the patients with pheochromocytomas may present with normal blood pressure (BP) levels. Nonetheless, in general the most accepted prevalence for normotensive pheochromocytomas is 30% (6-8).

Despite the relative high frequency of normotensive pheochromocytomas according to some series, few data exist about the postoperative outcomes of these patients in comparison with hypertensive pheochromocytomas. In fact, few series have compared the risk of intraoperative and postoperative complications in both groups (8-11), and results are contradictory. Some authors have found a similar risk of intraoperative and postoperative complications in hypertensive and normotensive pheochromocytomas (10), while others described a lower risk in normotensive patients (8); even more, some series have reported a greater risk of complications when α-blockade is administered to normotensive patients (11, 12). There is a lack of evidence about the benefits of presurgical preparation in normotensive pheochromocytomas.

Considering this background, the aim of this study was to analyze differences in postoperative outcomes following adrenalectomy in patients with normotensive and hypertensive pheochromocytomas. We also evaluated the effect of hypertension after controlling the demographic differences in both groups using multivariable logistic analysis.

## Material and Methods

### Study Design

This study is an international, multicenter retrospective cohort study evaluating patients diagnosed with pheochromocytoma who underwent surgery during the 10-year period from January 2012 to December 2022. Data were collected from 46 centers in 20 countries across 4 continents—centers were approached for inclusion in this study if they had published on the topic during the 10 years of this study. At the lead center (Queen Elizabeth Hospital, Birmingham, UK), the study was approved as a clinical audit, with registration CARMS-18769. In addition, the ethical committee of the Ramón y Cajal Hospital approved the study (N REF 101/21, approval date: December 20, 2022, ACTA 445). In other countries, local principal investigators were responsible for registering this study with the local committee.

### Study Population, Demographic, and Outcome Definitions

Patients included in the study had a preoperative pheochromocytoma diagnosis confirmed or suspected based on cross-sectional imaging (computed tomography or magnetic resonance imaging) combined with elevated plasma/urine metanephrines levels according to individual center protocols.

**Figure 1.**
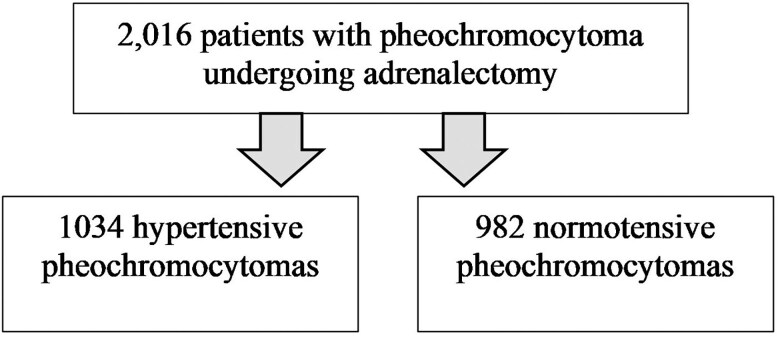
Study population.

In addition, confirmed histological diagnosis of pheochromocytoma was also required. As we have previously defined in the PHEO-Risk study (8), normotensive pheochromocytoma was defined as a patient with no previous history or any episode of hypertension (office systolic blood pressure [SBP] > 140 mm Hg or office diastolic blood pressure [DBP] > 90 mm Hg) and no use of any type of antihypertensive medications before surgery; and hypertensive pheochromocytoma when office SBP was greater than 140 mm Hg and/or office DBP was greater than 90 mm Hg before surgery, or the patient was under medical treatment with antihypertensive drugs (Fig. 1). Of the 2016 patients with pheochromocytoma undergoing adrenalectomy included, 1034 had preoperative hypertension and 982 were classified as normotensive pheochromocytomas.

Demographics collected for this study included age, sex, body mass index (BMI), and preoperative comorbidities. Preoperative comorbidities were scored according to the Charlson Comorbidity Index (13), and physical status was assessed using the American Society of Anesthesiologists (ASA) score (14). We also collected data on tumor characteristics including tumor laterality, size, and underlying genetic conditions. For tumor size, diameter of the largest lesion was used in cases of bilateral tumors. Surgical approach was characterized as open approach (OA), laparoscopic approach (LA), or robotic approach (RA). Minimally invasive approaches were completed using either transperitoneal (TPA) or retroperitoneal (RPA) techniques. Operative time was defined as the time between skin incision and skin closure. Conversion to open was defined as the need for laparotomy during RA or LA or completing the surgery with hand-assisted (HA) technique.

Perioperative complications and postoperative outcomes were evaluated. Complications were considered as “any deviation from the normal postoperative course,” according to the Clavien-Dindo Classification (CDC) (15). Each complication was assigned a CDC grade between I and V, based on the degree of invasiveness of the required treatment, with grade V representing death. The Comprehensive Complication Index (CCI) was used to score (0-100) the overall burden of complications within surgery and discharge (16). Any new complication and the relative CCI were calculated at discharge, 90 days, and 12 months. Severe complications were defined as a CDC greater than or equal to 3a. Length of hospital stay (LOS) was calculated from the day of the operation until the day of discharge. Of note, prolonged vasopressor support, defined as postoperative hypotension requiring vasopressor support for more than 24 hours, was considered as a grade IVa complication based on previous studies (17, 18). Readmission rates to hospital at 90 days for any causes (surgical or medical) were evaluated. Obesity was defined as BMI of 30 or greater at surgery.

The primary outcome of this study was to evaluate the effect of preoperative hypertension on postoperative outcomes following adrenalectomy for pheochromocytoma. Secondary outcomes evaluated differences in patients with hypertension and those without.

### Statistical Analysis

Data were presented as median with interquartile range (IQR) for continuous variables and differences between groups evaluated using bivariate regression. Data for CCI are presented as mean ± SD due to all medians being 0. Categorical variables were presented as counts and percentages, with differences analyzed using chi-square tests. Results from multivariable analysis were presented as odd ratios (OR) and outcomes following propensity score matching were presented as average effect on the treated cohort with 95% CI. The α was set to *P* less than .05 a priori.

Initially, nonadjusted bivariate analyses of demographics and outcomes were conducted comparing hypertensive and nonhypertensive cohorts. To control for demographic differences, multivariable models were constructed to evaluate factors (including our factor of interest, hypertension) for independent association with complications and CCI. To develop these models, factors statistically significantly associated with outcomes with a *P* value of less than .10 were incorporated in the main effects logistic regression model. The Wald test was used to determine statistically significant variables (*P* < .05) contributing to the model. Collinearity was evaluated using the variance inflation factor, with greater than 10 prompting exclusion from the model. The Brier score (BS) and receiver operating characteristic (ROC) curves were used to assess goodness of fit of the models. Analysis was conducted using Stata 17 (STATA Corp LP).

To further control for demographic differences and evaluate outcomes a propensity score matched (PSM) analysis was completed. Factors determined to be statistically significant in the multivariable analysis were controlled for in addition to any demographic factors deemed clinically relevant. Hypertensive and nonhypertensive patients were matched via PSM using probit treatment modeling controlling for surgical approach, age, sex, Charlson Comorbidity index, tumor size, tumor location, whether a retroperitoneal approach was applied, and whether patients received α- or β-blockers preoperatively. Replacements were allowed to optimize matching, meaning hypertensive patients could match with nonhypertensive patients more than once. Following PSM, we assessed outcomes including the CCI at discharge, CCI at 90 days, CCI at 1 year, readmission rates, operative time, estimated blood loss, total LOS, occurrence of any complication, serious complications, serious complications excluding hypotension, and episodes of hypotension. Subgroups analyses comparing sporadic and genetic tumors were performed.

## Results

### Baseline Characteristics

This study included 2016 patients with pheochromocytoma undergoing adrenalectomy. Of these, 1034 (51.2%) had preoperative hypertension and 982 (49.8%) were classified as normotensives. The majority of patients underwent LA (81.3%), and most procedures were TPA (67.2%; Table 1). Patients had a median age of 52.0 years and 45.0% were female. The median BMI was of 25.0. In terms of comorbidities, the median Charlson Comorbidity Index was 2.0, and most patients had an ASA of II or III (59.5% and 28.7%, respectively). The median tumor size was 4.0 cm, and there was an equal distribution of right- and left-sided tumors (see Table 1). Overall, the practice for admitting patients before surgery for fluid hydration varied across centers. Of included centers, 10 centers had no patients admitted preoperatively for intravenous fluid hydration, while 13 centers admitted all of their included patients preoperatively for fluid hydration. The remaining centers had between 1.5% and 98.3% of patients admitted preoperatively for intravenous fluid hydration. Of patients, 705 (37.9%) received preoperative admission for intravenous fluid hydration. There was no statistically significant difference in preadmission between the hypertensive and nonhypertensive cohorts (371, 37.2% hypertensive vs 334, 38.8% nonhypertensive; *P* = .533). Importantly, 93.2% of patients received α-blockade with a similar proportion having blockade discontinued the day of surgery (45.3%) and more than 10 days before surgery (49.6%). Only 32.5% of patients also had β-blockade. Detailed information regarding α- and β-blocker molecules and dosage are presented Tables 2 and 3. In summary, when we compared patients with and without α-blockade, we found that patients without α-blockade were more likely to undergo open surgery (14.4% vs 10.9%; *P* = .024) and more likely to have a TPA (82.2% vs 65.8%; *P* < .001). Moreover, they had a significantly lower starting mean arterial pressure, and they were less likely to require the intensive care unit (ICU) and required fewer blood transfusions. They also had a shorter LOS and significantly fewer complications (10.1% vs 20.6%; *P* < .001) and were much less likely to require vasopressors at closure (6.9% vs 18.6%; *P* < .001). Incidental diagnosis was more frequent in the nonhypertensive group (56.4% vs 32.5%; *P* < .001) (see Table 1).

**Table 1.** Baseline characteristics of the population in the international pheochromocytoma database comparing hypertensive and nonhypertensive patients

		Unmatched cohort			Propensity matched cohort		
	Total cohort n = 2016	Nonhypertensive n = 982	Hypertensive n = 1034	*P*	Nonhypertensive n = 248	Hypertensive n = 1024	*P*
Surgical approach				.376			.966
Open	221 (11.1%)	112 (11.7)	109 (10.6)		28 (11.3)	106 (10.4)	
Laparoscopic	1,613 (81.3)	779 (81.7)	834 (80.9)		198 (79.8)	830 (81.1)	
Robotic	142 (7.2)	59 (6.2)	83 (8.1)		21 (8.5)	83 (8.1)	
Hand assisted	9 (0.5)	4 (0.4)	5 (0.5)		1 (0.4)	5 (0.5)	
Age, y	52.0 (41.0-63.0)	50.0 (39.0-61.0)	54.5 (44.0-65.0)	<.001	50.0 (40.0-61.0)	54.5 (44.0-65.0)	.849
BMI	25.0 (22.4-28.1)	25.0 (22.0-28.0)	25.0 (23.0-28.5)	.090	25.3 (23.0-29.0)	25.0 (23.0-28.5)	.574
Female	908 (45.0)	445 (45.3)	463 (44.8)	.808	117 (47.2)	459 (44.8)	.504
Charlson index	2.0 (1.0-3.0)	1.0 (0.0-2.0)	2.0 (1.0-4.0)	<.001	2.0 (0.0-2.0)	2.0 (1.0-4.0)	<.001
ASA score				<.001			<.001
1	204 (10.1)	158 (16.1)	46 (4.5)		32 (12.9)	46 (4.5)	
2	1,198 (59.5)	628 (64.0)	564 (54.6)		162 (65.3)	561 (54.8)	
3	577 (28.7)	189 (19.3)	388 (37.6)		52 (21.0)	382 (37.3)	
4	41 (2.0)	6 (0.6)	35 (3.4)		2 (0.8)	35 (3.4)	
Comorbidities				<.001			<.001
Type 2 diabetes	407 (20.2)	117 (11.9)	290 (28.0)		28 (11.3)	288 (28.1)	
Hypertension	744 (64.6)	0 (0)	1034 (100)		0 (0)	1024 (100)	
Diagnosed as incidental finding	692 (42.2)	376 (56.4)	316 (32.5)	<.001	102 (59.0)	310 (32.2)	.001
Indication for resection							
Elevated catecholamine or symptoms	1015 (63.4)	381 (57.8)	634 (67.3)	<.001	91 (52.9)	631 (67.6)	.002
Imaging suspected malignancy	501 (31.3)	232 (35.2)	269 (28.6)		67 (39.0)	263 (28.2)	
Tumor size	65 (4.1)	37 (5.6)	28 (3)		10 (5.8)	28 (3.0)	
Metastasis	2 (0.1)	0 (0)	2 (0.2)		0 (0)	2 (0.2)	
Other	18 (1.1)	9 (1.4)	9 (1.0)		4 (2.3)	9 (1.0)	
Genetic condition							
Yes, n (%)	286 (14.2)	161 (16.4)	125 (12.1)	.006	49 (19.8)	123 (12.0)	.001
Approach				.408			
Transperitoneal	1,327 (67.2)	624 (65.8)	703 (68.5)		153 (61.7)	700 (68.6)	.105
Retroperitoneal	609 (30.8)	303 (32.0)	306 (29.8)		89 (35.9)	303 (29.7)	
Other	39 (2.0)	21 (2.2)	18 (1.8)		6 (2.4)	17 (1.7)	
Tumor size, largest tumor cm	4.0 (3.0-5.8)	4.0 (3.0-5.8)	4.2 (3.0-5.9)	.318	4.0 (2.7-5.5)	4.1 (3.0-6.0)	.542
Tumor location				.191			
Right	959 (48.9)	477 (50.9)	482 (47.1)		136 (56.7)	479 (47.2)	
Left	934 (47.7)	432 (46.1)	502 (49.1)		99 (41.3)	498 (49.1)	.022
Bilateral	67 (3.4)	28 (3.0)	39 (3.8)		5 (2.1)	38 (3.7)	
α-Blockade							
Yes	1,833 (93.2)	878 (93.2)	955 (93.2)	.975	229 (92.7)	947 (93.2)	.782
α-Blocker discontinuation, (days)				<.001			<.001
0	952 (45.3)	383 (35.8)	569 (55.0)		100 (40.3)	564 (55.1)	
1-10	109 (5.2)	49 (4.6)	60 (5.8)		14 (5.7)	59 (5.8)	
>10	1,042 (49.6)	637 (59.6)	405 (39.2)		134 (54.0)	401 (39.2)	
β-Blockade	650 (33.8)	264 (28.5)	386 (38.9)	<.001	74 (30.3)	385 (39.1)	.011

Abbreviations: ASA, American Society of Anesthesiologists; BMI, body mass index.

**Table 2.** α-Blockade type and dosage compared between the two cohorts

α-blockade	Total cohort n = 2016	Nonhypertensive n = 982	Hypertensive n = 1034	*P*
Phenoxybenzamine, n (%)	962 (53.8)	552 (64.6)	410 (43.9)	
Daily dose, mg	40 (20-60)	40 (20-60)	40 (20-60)	<.001*^a^*
Doxazosin, n (%)	727 (40.6)	276 (32.3)	451 (48.2)	
Daily dose, mg	6 (4-12)	4 (4-10)	6 (4-12)	
Prazosin, n (%)	57 (3.2)	15 (1.8)	42 (4.5)	
Daily dose, mg	3.75 (2.5-5)	2.75 (1-5)	4 (2.5-5)	
Terazosin, n (%)	6 (0.3)	3 (0.4)	3 (0.3)	
Daily dose, mg	4 (1-8)	4 (1-8)	4 (1-16)	

Data are presented with median and interquartile range (IQR) for daily dose. The selection of preoperative drug, dosage, and treatment of α-/β-blockade was based on centers’ protocols. Of all patients, 1833 (93.2%) received α-blockade with similar rates between the hypertensive (93.2%) and nonhypertensive (93.2%) cohorts (*P* = .975). The most commonly used molecules for α-blockade were phenoxybenzamine (962 patients), doxazosin (727 patients), prazosin (57 patients), and terazosin (6 patients) with a median daily dose of 40 (IQR 20-60), 6 (4-12), 3.75 (IQR 2.5-5), 4 (IQR 1-8) mg, respectively. The remaining 81 patients used other drugs or combination; however, detailed information on this was missing.

^a^ *P* value represents χ^2^ analysis comparing rates of α-blocker drug types as a whole between hypertensive and nonhypertensive patients.

**Table 3.** β-Blockade type and dosage compared between the two cohorts

β-blockade	Total cohort n = 2016	Nonhypertensive n = 982	Hypertensive n = 1034	*P*
Propranolol, n (%)	197 (30.8)	112 (43.1)	85 (22.4)	<.001*^a^*
Daily dose, mg	30 (20-60)	30 (20-60)	30 (20-60)	
Metoprolol, n (%)	146 (22.8)	30 (11.5)	116 (30.5)	
Daily dose, mg	50 (25-100)	50 (25-100)	50 (25-100)	
Bisoprolol, n (%)	121 (18.9)	46 (17.7)	75 (19.7)	
Daily dose, mg	2.5 (2.5-5)	2.5 (1.9-5)	2.5 (2.5-5)	
Atenolol, n (%)	81 (12.6)	41 (15.8)	50 (10.5)	
Daily dose, mg	50 (25-50)	50 (25-50)	50 (25-50)	
Carvedilol, n (%)	36 (5.6)	11 (4.2)	25 (6.6)	
Daily dose, mg	25 (12.5-50)	25 (12.5-50)	25 (12.5-25)	
Labetalol, n (%)	12 (1.9)	1 (0.4)	11 (2.9)	
Daily dose, mg	300 (100-600)	400 (400-400)	200 (100-600)	

Data are presented with median and interquartile range for daily dose. In terms of β-blockade, 650 (33.9%) patients received preoperative treatment with significantly more hypertensive patients (38.9%) requiring therapy than nonhypertensive (28.5%). Looking at the β-blockade molecules, the most commonly used ones were propranolol (197 patients), metoprolol (146 patients), bisoprolol (121 patients), atenolol (81 patients), carvedilol (36 patients), and labetalol (12 patients) with a median daily dose of 30 (20-60), 50 (25-100), 2.5 (2.5-5), 50 (25-50), 25 (12.5-50), and 300 (100-600) mg, respectively. The remaining 57 patients used other drugs or combination; however, detailed information on this was missing.

^a^ *P* value represents χ^2^ analysis comparing rates of β-blocker drug types as a whole between hypertensive and nonhypertensive patients.

Comparatively, hypertensive patients were 4.5 years older (*P* < .001), had a higher prevalence of type 2 diabetes (*P* < .001) and had a higher median Charlson Comorbidity Index (2.0 vs 1.0; *P* < .001) and higher ASA. In addition, normotensive patients had hereditary syndromes associated with pheochromocytomas more commonly than hypertensive patients (16.1% vs 12.5%; *P* = .006). Elevated catecholamines or symptoms was the most common indication for surgery and occurred more frequently in hypertensive patients (67.3% vs 57.8%; *P* < .001). The second most common indication for resection was imaging suspected malignancy, which was more likely to occur in nonhypertensive patients (35.2% vs 28.6%; *P* < .001) (see Table 1). Operative approaches, tumor size, tumor laterality, and the proportion of patients with α-blockade was similar between cohorts. Notably, patients with hypertension were more likely to continue their α-blockade until the day of surgery (55.0% vs 35.8%; *P* < .001; see Table 1) and were more likely to have β-blockade (38.9% vs 28.5%; *P* < .001).

### Bivariate Analysis of Postoperative Outcomes

Nonadjusted outcome analysis demonstrated that patients with hypertension had a longer operative duration (115.0 vs 103.5 minutes; *P* = .026) and had a higher rate of vasopressors at skin closure (19.7% vs 15.4%; *P* = .013). Additionally, patients with hypertension had a higher rate of perioperative blood transfusion (7.7% vs 5.0%; *P* = .016; Table 4). In keeping with these findings, patients with hypertension had a higher rate of any complication during their hospital stay (21.6% vs 17.7%; *P* = .029). However, with regard to conversion, readmissions, postoperative hypotension within and beyond 24 hours, and summative measures of morbidity including serious complications and CCI, the cohorts were similar (see Table 4).

**Table 4.** Clinical outcomes compared between hypertensive and nonhypertensive patients undergoing adrenalectomy

	Total cohort n = 2016	Nonhypertensive n = 982	Hypertensive n = 1034	*P*
Postoperative location				<.001
Ward	851 (43.2)	432 (46.0)	419 (40.7)	
ICU	742 (37.7)	312 (33.2)	430 (41.8)	
Step-Down unit	375 (19.1)	195 (20.8)	180 (17.5)	
Operative time, min	110 (75.0-157.0)	103.5 (75.0-150.0)	115.0 (78.0-161.0)	.026
Estimated blood loss, mL	20.0 (0.0-100.0)	20.0 (0.0-100.0)	20.0 (10.0-100.0)	.295
Vasopressor at skin closure	336 (17.7)	135 (15.4)	201 (19.7)	.013
Perioperative blood transfusion	123 (6.4)	45 (5.0)	78 (7.7)	.016
Converted to open/HA	73 (3.6)	35 (3.6)	38 (3.7)	.894
Reason for conversion				.521
Bleeding	32 (1.6)	16 (1.6)	16 (1.6)	
Instability	9 (0.5)	3 (0.3)	6 (0.6)	
Tumor size	10 (0.5)	3 (0.3)	7 (0.7)	
Other	22 (1.1)	13 (1.3)	9 (0.9)	
Postoperative hypotension	369 (23.0)	170 (23.1)	199 (22.9)	.652
Postoperative hypotension beyond 24 h	101 (6.4)	52 (7.7)	49 (5.4)	.062
Any complications within hospital stay	392 (19.8)	169 (17.7)	223 (21.6)	.029
Length of hospital stay, days	4.0 (3.0-7.0)	4.0 (3.0-7.0)	4.0 (3.0-7.0)	.051
CCI				
At discharge	5.6 (12.9)	5.1 (12.4)	6.1 (13.4)	.077
90 days	5.9 (13.3)	5.3 (12.6)	6.4 (13.9)	.066
12 months	6.0 (13.4)	5.4 (12.6)	6.6 (4.2)	.040
Severe complications CDC ≥3a	135 (6.8)	68 (7.0)	67 (6.6)	.722
Severe complication excluding hypotension	37 (1.9)	17 (1.8)	20 (2.0)	.717
Readmission at 90 days, n (%)	34 (1.7)	14 (1.4)	20 (1.9)	.381
Postoperative mortality (until hospital discharge)	0	0	0	—

Data are presented as median and interquartile range for continuous variables.

Abbreviations: CCI, Comprehensive Complication Index; CDC, Clavien-Dindo classification; HA, hand assisted; ICU, intensive care unit.

### Demographic-Adjusted Postoperative Outcomes

After controlling for demographic differences with multivariable logistic analysis, hypertension was not independently associated with complications (OR 1.16; *P* = .260; Table 5). Completing the surgery laparoscopically or robotically (compared to open) was independently associated with a reduced likelihood of complications. On the other hand, having β- (OR 1.37; *P* = .018) or α (OR 1.97; *P* = .042) blockade independently increased the likelihood of complications (see Table 5). The model was accurate with an ROC of 0.688 and BS of 0.144.

**Table 5.** Multivariable logistic regression for complications and multivariable linear regression for Comprehensive Complication Index at time of discharge for all patients undergoing adrenalectomy

Multivariable logistic regression evaluating factors associated with any complication			
Risk factor	Odds ratio	95% CI	*P*
Hypertension	1.16	0.90 to 1.49	.260
Surgical approach, compared to open			
Laparoscopic	0.34	0.24 to 0.49	<.001
Robotic	0.37	0.21 to 0.67	.001
Hand assisted	1.40	0.32 to 6.12	.653
Age	0.99	0.98 to 1.00	.208
Sex, female compared to male	1.10	0.86 to 1.41	.432
Charlson comorbidity index	1.12	1.03 to 1.22	.008
Tumor size, cm	1.14	1.09 to 1.19	<.001
Tumor laterality			
Left-sided tumor, compared to right	1.00	0.79 to 1.29	.954
Bilateral tumor, compared to right	1.11	0.58 to 2.13	.753
Retroperitoneal approach, compared to transperitoneal	1.03	0.82 to 1.31	.789
β-Blockade	1.37	1.06 to 1.79	.018
α-Blockade	1.97	1.09 to 3.81	.042
ROC: 0.688; BS: 0.144			

Multivariable linear regression evaluating factors associated with increasing Comprehensive Complication Index at discharge			
Risk factor	Coefficient	95% CI	*P*
Hypertension	0.20	−0.98 to 1.39	.734
Surgical approach, compared to open			
Laparoscopic	−7.71	−9.75 to −5.66	<.001
Robotic	−7.62	−10.60 to −4.74	<.001
Hand assisted	6.10	−2.76 to 14.84	.174
Age	−0.03	−0.08 to 0.02	.315
Sex, female compared to male	1.00	−0.12 to 2.16	.083
Charlson Comorbidity Index	0.52	0.12 to 0.96	.015
Tumor size, cm	0.81	0.58 to 1.05	<.001
Tumor laterality			
Left-sided tumor, compared to right	0.39	−0.76 to 1.54	.509
Bilateral tumor, compared to right	0.18	−3.08 to 3.44	.913
Retroperitoneal approach, compared to transperitoneal	−0.64	−1.78 to 0.50	.270
β-Blockade	1.66	0.38 to 2.93	.011
α-Blockade	2.57	0.01 to 5.13	.049
*R* ^2^: 0.109			

Abbreviations: BS, Brier score; ROC, receiver operating characteristic.

Similarly, multivariable logistic regression evaluating factors independently affecting CCI demonstrated similar results (see Table 5). Again, a minimally invasive approach was associated with a lower CCI and having β- (1.65; *P* = .011) or α- (2.57; *P* = .049) blockade was independently associated with an increased CCI. Additionally, in this model a higher Charlson Comorbidity Index and larger tumor size was also associated with increasing CCI (see Table 5).

Finally, comparing hypertensive and normotensive patients following 1:1 PSM demonstrated that CCI at all time points, readmission, complications, and serious complications were similar between cohorts (Table 6). Additionally, patients had a similar rate of hypotension episodes within and beyond 24 hours (see Table 6). The sole difference noted following PSM was that patients with hypertension had a 0.83 (95% CI, 0.31-1.36) day increased LOS (*P* = .002).

**Table 6.** Comparation of surgical outcomes in hypertensive pheochromocytomas compared to normotensive pheochromocytomas undergoing adrenalectomy following 1:1 propensity score matching

Outcome	Average estimated treatment effect	95% CI	*P*
CCI at discharge	1.00	−0.79 to 2.78	.274
CCI at 90 days	0.92	−0.91 to 2.75	.324
CCI at 1 year	1.09	−0.74 to 2.91	.242
Readmission at 90 days	−0.1%	−2.2% to 2.0%	.924
Length of stay, days	0.83	0.31 to 1.36	.002
Any complication	4.7%	−0.6% to 10.1%	.086
Serious complication	−0.4%	−4.2% to 3.4%	.829
Serious complication excluding hypotension	0.9%	−0.5% to 2.4%	.210
Hypotension, <90 mm Hg within 24 h postoperatively	−3.2%	−8.9% to 2.5%	.265
Hypotension, <90 mm Hg beyond 24 h postoperatively	−3.8%	−7.7% to 0.2%	.062

Abbreviation: CCI, Comprehensive Complication Index.

### Subgroup Analyses of Genetic Syndromes and Sporadic Pheochromocytoma

Due to the differences in rates of sporadic tumors, further subgroup analysis was also conducted comparing hypertensive and normotensive patients in patients after excluding those with genetic syndromes. In this analysis there were 1728 patients evaluated, with 820 (47.3%) not having hypertension and 908 (52.6%) being hypertensive. Multivariable modeling demonstrated similar findings with hypertension not being independently associated with complication occurrences (Table 7).

**Table 7.** Multivariable logistic regression for complications at time of discharge for patients undergoing adrenalectomy with sporadic tumors (ie, excluding those with genetic syndromes)

Multivariable logistic regression evaluating factors associated with any complication			
Risk factor	Odds ratio	95% CI	*P*
Hypertension	1.22	0.93-1.61	.149
Surgical approach, compared to open			
Laparoscopic	0.35	0.23-0.52	<.001
Robotic	0.34	0.18-0.64	.001
Hand assisted	1.30	0.29-5.78	.728
Age	0.99	0.98-1.00	.089
Sex, female compared to male	1.12	0.86-1.45	.399
Charlson Comorbidity Index	1.13	1.03-1.24	.013
Tumor size, cm	1.15	1.10-1.21	<.001
Tumor laterality			
Left-sided tumor, compared to right	0.96	0.74-1.24	.732
Bilateral tumor, compared to right	1.12	0.36-3.46	.847
Retroperitoneal approach, compared to transperitoneal	0.99	0.77-1.27	.928
β-Blockade	1.48	1.11-1.97	.007
α-Blockade	1.88	0.93-3.79	.077
ROC: 0.688; BS: 0.144			

Abbreviations: BS, Brier score; ROC, receiver operating characteristic.

However, following 1:1 propensity matched analysis of patients with sporadic tumors, some unique findings were noted. Interestingly, patients with hypertension had a small but significantly higher CCI at all time points (Table 8). Additionally, they were more likely to experience any complication (+6.6%; 95% CI, 1.9%-11.3%) and serious complications when excluding hypotensive episodes (+1.3%; 95% CI, 0.1%-2.6%).

**Table 8.** Comparation of surgical outcomes in hypertensive pheochromocytomas compared to normotensive pheochromocytomas undergoing adrenalectomy following 1:1 propensity score matching after exclusion of patients with genetic syndrome-linked tumors

Outcome	Average estimated treatment effect	95% CI	*P*
CCI at discharge	1.57	0.06 to 3.09	.042
CCI at 90 days	1.74	0.18 to 3.29	.026
CCI at 1 year	1.77	0.21 to 3.33	.029
Readmission at 90 days	0.1%	−1.6% to 1.3%	.875
Length of stay, days	0.60	0.06 to 1.15	.030
Any complication	6.6%	1.9% to 11.3%	.006
Serious complication	−1.3%	−5.3% to 2.7%	.532
Serious complication excluding hypotension	1.3%	0.1% to 2.6%	.047
Hypotension, <90 mm Hg within 24 h postoperatively	−2.3%	−7.9% to 3.3%	.418
Hypotension, <90 mm Hg beyond 24 h postoperatively	−2.2%	−6.9% to 2.3%	.331

Abbreviation: CCI, Comprehensive Complication Index.

## Discussion

Clinical diagnosis and treatment of hypertensive and normotensive pheochromocytomas is still a matter of debate. Despite current clinical guidelines that suggest a similar approach in both cases (2), some differences, related to surgical complications and treatment tolerance, are observed in clinical practice. In this context, comprehensive analyses of large cohorts may help to differentiate tumor subgroups and personalize clinical management.

This is the largest cohort that evaluates the effect of hypertension on postoperative outcomes in patients with pheochromocytomas. We observed that patients with hypertension had a higher risk of any postoperative complication than normotensive individuals, but these differences were related to a higher ASA, Charlson Comorbidity index, and older age; postoperative outcomes were similar in both groups after controlling for demographic differences and following 1:1 PSM, except for a longer LOS in the group of hypertensive pheochromocytoma patients.

Regarding the prevalence of normotension in patients with pheochromocytomas, we found that up to 50% were classified as normotensive cases. This rate is higher than in other series, in which the prevalence ranges between 6% (4) and 20% to 30% (6); differences might be related to the definition of hypertension used at different centers and the bias of retrospective studies. Importantly, incidence rates are larger in series of incidental pheochromocytomas, ranging from 13.5% to 55% (4, 5). In this context, a German cohort of 201 pheochromocytomas, which used the same definition for hypertension as this study, reported only 6.1% of normotensive cases, but the typical triad of symptoms (headache, palpitations, and sweating) was found in only 10% of cases (4). Importantly, the pattern of hypertension is widely variable in these patients, for example, in the German cohort, 8.2% presented only with BP peaks and no evidence of permanent hypertension, 35.9% had persistent hypertension with BP peaks, and 55.9% presented with hypertension without BP peaks. Additionally, although data on ambulatory BP monitoring (ABPM) in pheochromocytoma patients are limited, some studies suggest that around 10% of cases with normal office BP levels are hypertensive based on the results of ABPM (19). In this regard, despite a lack of ABPM data in our cohort, probably some normotensive patients in our cohort may have had undiagnosed hypertension. Furthermore, the variability of BP throughout the day and its circadian rhythm are associated with clinical complications, specifically, a greater variability and the loss of nighttime BP dip are associated with increased target organ damage. In this sense, pheochromocytomas and sympathetic paragangliomas (PPGLs) with inverted circadian rhythm have higher BP variability, which might be closely associated with hormone secretion since it reversed after tumor removal (20). Moreover, pheochromocytoma can also present with orthostatic hypotension due to volume depletion and loss of baroreceptor sensitivity (21). All these data suggest that pheochromocytoma should be considered independent of the presence of hypertension, especially when atypical signs or symptoms are present, such as with atypical diabetes mellitus or takotsubo syndrome (22).

In this cohort, hypertensive patients were older and had a higher median Charlson Comorbidity Index and ASA than the normotensive patients. These results are in line with the results of the PHEO-Risk study, in which a difference of approximately 10 years was observed between patients with normotensive and hypertensive pheochromocytomas; furthermore, a higher prevalence of comorbidities including diabetes mellitus and cardiovascular disease was observed in patients with hypertension (8), which is consistent with higher catecholamine secretion (23). In this context, it is possible that the higher comorbidity burden in these hypertensive patients is partially explained by their age. Nonetheless, it is known that patients with hypertensive pheochromocytomas have higher catecholamines levels than normotensive patients, leading to a higher risk of cardiovascular complications (24). In this regard, the clinical phenotype of hypertension depends on multiple factors including adrenal content of catecholamines, as well as the pattern and nature of the secretion. For example, persistent hypertension strongly correlates with high levels of continuously released plasma norepinephrine, while paroxysmal hypertension occurs mostly in patients with high levels of plasma epinephrine (24). We also found that normotensive pheochromocytomas occurred more commonly in patients with hereditary conditions. These results are in line with those reported in the PHEO-Risk study, in which we found that the prevalence of hereditary syndromes was 2-fold higher in patients with normotensive than hypertensive pheochromocytomas (26.8% vs 56.3%; < 0.001) (8). The association between normotensive pheochromocytomas and hereditary syndromes may be explained by an earlier diagnosis of the disease in these patients, leading to the detection of smaller tumors with lower secretion of catecholamines.

Clinical presentation in these tumors is important not only for diagnosis, but also for preoperative medical treatment. In this sense, current guidelines suggest a similar approach in patients with and without hypertension (7); in this cohort we observed that patients with hypertension were more likely to continue their α-blockade until the day of surgery, and were more likely to require β-blockade. Similar results were described in previous Spanish series, in which a higher proportion of patients with normotensive PPGLs did not receive presurgical α-blockade compared with hypertensive PPGLs (12.6% vs 4.3%; *P* = .009) (8). This therapeutic attitude may be conditioned by some studies that have described a high risk of cardiovascular collapse with α-blockade in dopamine-secreting PPGLs (12). However, this is a report of only 2 dopamine-secreting pheochromocytomas; thus, larger series are needed to establish a recommendation against the use of α-blockers in normotensive PPGLs.

Despite normotensive incidental pheochromocytomas having similar radiological and morphological characteristics compared to hypertensive pheochromocytomas, these tumors are biochemically different, with reduced levels of urinary epinephrine and norepinephrine (6); thus, a different rate of complications would be expected. Specifically, low amounts of circulating catecholamines and intermittent release are associated with weaker clinical expression (24, 25). The underlying mechanisms for these clinical differences are still unknown, and might be related to the downregulation of some genes involved in key processes of catecholamine metabolism (including the tyrosine hydroxylase enzyme) in nonhypertensive pheochromocytomas (6).

In this cohort, after controlling for demographic differences with multivariable logistic analysis, hypertension was not independently associated with complications; only in hypertensive patients was a slightly increased LOS observed. As in our study, other authors report no differences in the prevalence of serious cardiovascular complications in patients with hypertensive and normotensive tumors after adjusting for confounding factors (26); furthermore, silent tumors with slight organ damage (even in older patients) have been reported (5) and the per-operative hemodynamic instability is similar in both groups in other series (10). The observed association of hypertension with older age and other comorbidities is in line with several reports; specifically, observational studies in frail older patients with hypertension have higher morbidity and mortality rates compared with those with lower BP levels; furthermore, hypertension has considerable effects on cardiovascular effects including heart failure, myocardial infarction, and stroke (27). Additionally, hypertension increases in parallel with age, increasing from 27% in patients younger than 60 years to 74% in those older than 80 years (28). In this sense, the Framingham Heart Study showed that more than 90% of the participants with a normal BP at age 55 years eventually develop hypertension (29).

In contrast to our results, higher rates of renal failure in normotensive patients have been also described (5); furthermore, insufficient BP control before surgery was associated with a higher risk of intraoperative complications (30), while in patients with nonhypertensive tumors, similar heart rate and BP have been reported independent of α-blocker treatment (31). In this line, previous reports suggest that hypertensive and normotensive pheochromocytomas might be different entities based on the cellular chromaffin machinery (6), but described differences might be also explained by the sample size and nonadjusted analyses.

As previously described, we observed a lower risk of complications after laparoscopic or robotic surgery compared to open surgery. Supporting it, a meta-analysis including 14 studies involving 626 patients focused on comparing LA and OA found that the LA approach caused less intraoperative hemodynamic instability, providing an equal chance to cure hypertension while also yielding a faster and better postoperative recovery (32), with even reduced rates of severe complications with the RA (33). An unexpected result in our study was the increased likelihood of complications in patients under treatment with β- or α-blockade. These results could go against the current recommendation of using α-blockade preoperatively in all patients with pheochromocytomas (2), in line with some recent studies that defend an absent benefit of α-blockade in these patients (34, 35). Regarding this, in the meta-analysis of Schimmack et al (34), mortality, cardiovascular complications, mean maximal intraoperative SBP and DBP, and mean maximal intraoperative heart rate did not differ between patients with or without α-blockade. Furthermore, in the Groeben international study (35), the cardiovascular complication rate was higher in the group of patients receiving α-blockade than among patients without α-receptor blockade (5.9% vs 0.9%; *P* = .0006). However, results should be interpreted cautiously, since the authors did not describe side-by-side tumor characteristics of both groups; thus, differences in age, tumor size, and hormone secretion might have been present.

Although our study is the largest study evaluating the effect of hypertension on surgical outcomes, we are aware that it has some limitations, including its retrospective nature. Moreover, although the utilization of PSM was sought to limit this selection bias by controlling for important confounders, there could still be other unmeasured parameters and complications lost during data collection. Third, due to the multicenter nature of the study, we must consider that the population demographics differ considerably between centers, and this could have altered the decision-making processes and patient selection. For example, we found that hypertensive pheochromocytoma patients were transferred more frequently in the ICU, but we acknowledge that the reasons behind this may well reflect the different policies and protocols of patient management at each center; therefore, these results warrant cautious interpretation. We also must consider that the hypertension definition was based on ambulatory BP levels, so it is possible that the proportion of hypertensive cases has been underestimated. Moreover, detailed information on catecholamines levels is lacking; thus, it was not possible to evaluate the effect of the degree of catecholamine hypersecretion on surgical outcomes.

## Conclusion

Patients with hypertensive pheochromocytomas have a higher risk of postoperative complications than normotensive patients due to the association of hypertension with a higher burden of comorbidities and older age. However, hypertension is not an independent risk factor for postoperative complications in pheochromocytoma surgery since the risk of complications is similar in the group of hypertensive and normotensive pheochromocytomas after adjusting for demographic differences.

## Data Availability

The data that support the findings of this study are available on request from the corresponding authors.

## Abbreviations

ABPM: ambulatory blood pressure monitoring
ASA: American Society of Anesthesiologists
BMI: body mass index
BP: blood pressure
CCI: Comprehensive Complication Index
CDC: Clavien-Dindo classification
DBP: diastolic blood pressure
HA: hand assisted
ICU: intensive care unit
IQR: interquartile range
LA: laparoscopic adrenalectomy
LOS: length of hospital stay
OA: open adrenalectomy
OR: odds ratio
PPGLs: pheochromocytomas and sympathetic paragangliomas
PSM: propensity score matched
RA: robotic adrenalectomy
RPA: retroperitoneal
SBP: systolic blood pressure
TPA: transperitoneal approach

## References

[1] Araujo-Castro M . Pheochromocytoma. Preoperative approach. Med Clin (Barc). 2024;163(6):294‐300. 38849272 10.1016/j.medcli.2024.03.025

[2] Lenders JWM, Duh QY, Eisenhofer G, et al Pheochromocytoma and paraganglioma: an endocrine society clinical practice guideline. J Clin Endocrinol Metab. 2014;99(6):1915‐1942. 24893135 10.1210/jc.2014-1498

[3] Garcia-Carbonero R, Matute Teresa F, Mercader-Cidoncha E, et al Multidisciplinary practice guidelines for the diagnosis, genetic counseling and treatment of pheochromocytomas and paragangliomas. Clin Transl Oncol. 2021;23(10):1995‐2019. 33959901 10.1007/s12094-021-02622-9 PMC8390422

[4] Kopetschke R, Slisko M, Kilisli A, et al Frequent incidental discovery of phaeochromocytoma: data from a German cohort of 201 phaeochromocytoma. Eur J Endocrinol. 2009;161(2):355‐361. 19497985 10.1530/EJE-09-0384

[5] Noshiro T, Shimizu K, Watanabe T, et al Changes in clinical features and long-term prognosis in patients with pheochromocytoma. Am J Hypertens. 2000;13(1):35‐43. 10678269 10.1016/s0895-7061(99)00139-9

[6] Haissaguerre M, Courel M, Caron P, et al Normotensive incidentally discovered pheochromocytomas display specific biochemical, cellular, and molecular characteristics. J Clin Endocrinol Metab. 2013;98(11):4346‐4354. 24001749 10.1210/jc.2013-1844

[7] Lenders JWM, Kerstens MN, Amar L, et al Genetics, diagnosis, management and future directions of research of phaeochromocytoma and paraganglioma: a position statement and consensus of the Working Group on Endocrine Hypertension of the European Society of Hypertension. J Hypertens. 2020;38(8):1443‐1456. 32412940 10.1097/HJH.0000000000002438 PMC7486815

[8] Araujo-Castro M, García Sanz I, Mínguez Ojeda C, et al Differences in intraoperative and surgical outcomes between normotensive pheochromocytomas and sympathetic paragangliomas (PPGLs) and hypertensive PPGLs: results from the PHEO-RISK STUDY. J Endocrinol Invest. 2023;46(4):805‐814. 36323983 10.1007/s40618-022-01954-9

[9] Kong H, Li N, Li XY, Wang DX. The role of pre-operative α-blockade in patients with normotensive phaeochromocytoma or paraganglioma: a retrospective cohort study. Eur J Anaesthesiol. 2018;35(11):898‐899. 30278035 10.1097/EJA.0000000000000844

[10] Lafont M, Fagour C, Haissaguerre M, et al Per-operative hemodynamic instability in normotensive patients with incidentally discovered pheochromocytomas. J Clin Endocrinol Metab. 2015;100(2):417‐421. 25405501 10.1210/jc.2014-2998

[11] Foo SH, Chan SP, Ananda V, Rajasingam V. Dopamine-secreting phaeochromo-cytomas and paragangliomas: clinical features and management. Singapore Med J. 2010;51(5):e89‐e93. 20593136

[12] Dubois LA, Gray DK. Dopamine-secreting pheochromocytomas: in search of a syndrome. World J Surg. 2005;29(7):909‐913. 15951922 10.1007/s00268-005-7860-7

[13] Charlson ME, Pompei P, Ales KL, MacKenzie CR. A new method of classifying prognostic comorbidity in longitudinal studies: development and validation. J Chronic Dis. 1987;40(5):373‐383. 3558716 10.1016/0021-9681(87)90171-8

[14] Daabiss M . American Society of Anaesthesiologists physical status classification. Indian J Anaesth. 2011;55(2):111‐115. 21712864 10.4103/0019-5049.79879 PMC3106380

[15] Dindo D, Demartines N, Clavien PA. Classification of surgical complications: a new proposal with evaluation in a cohort of 6336 patients and results of a survey. Ann Surg. 2004;240(2):205‐213. 15273542 10.1097/01.sla.0000133083.54934.ae PMC1360123

[16] Slankamenac K, Graf R, Barkun J, Puhan MA, Clavien PA. The comprehensive complication index: a novel continuous scale to measure surgical morbidity. Ann Surg. 2013;258(1):1‐7. 23728278 10.1097/SLA.0b013e318296c732

[17] Parente A, Kamarajah SK, Thompson JP, et al Risk factors for postoperative complications after adrenalectomy for phaeochromocytoma: multicentre cohort study. BJS Open. 2023;7(5):zrad090. 37757753 10.1093/bjsopen/zrad090 PMC10533033

[18] Parente A, Thompson JP, Crook C, et al Risk factors for postoperative hypotension after adrenalectomy for phaeochromocytoma: derivation of the PACS risk score. Eur J Surg Oncol. 2023;49(2):497‐504. 36602554 10.1016/j.ejso.2022.10.006

[19] Memon SS, Srivastava P, Karlekar M, Thakkar H, Bandgar T. Ambulatory blood pressure monitoring in pheochromocytoma - paraganglioma: a single center experience. J Postgrad Med. 2024;70(2):84‐90. 37555422 10.4103/jpgm.jpgm_208_23 PMC11160991

[20] Zelinka T, Štrauch B, Petrák O, et al Increased blood pressure variability in pheochromocytoma compared to essential hypertension patients. J Hypertens. 2005;23(11):2033‐2039. 16208146 10.1097/01.hjh.0000185714.60788.52

[21] Bisogni V, Petramala L, Oliviero G, et al Analysis of short-term blood pressure variability in pheochromocytoma/paraganglioma patients. Cancers (Basel). 2019;11(5):658. 31083609 10.3390/cancers11050658 PMC6562701

[22] Araujo-Castro M, Mínguez Ojeda C, Garcia Centeno R, et al Glycemic disorders in patients with pheochromocytomas and sympathetic paragangliomas. Endocr Relat Cancer. 2022;29(12):645‐655. 36069783 10.1530/ERC-22-0218

[23] Stenstrom G, Sjostrom L, Smith U. Diabetes mellitus in phaeochromocytoma. Fasting blood glucose levels before and after surgery in 60 patients with phaeochromocytoma. Acta Endocrinol (Copenh). 1984;106(4):511‐515. 6475457 10.1530/acta.0.1060511

[24] Zuber SM, Kantorovich V, Pacak K. Hypertension in pheochromocytoma: characteristics and treatment. Endocrinol Metab Clin North Am. 2011;40(2):295‐311. 21565668 10.1016/j.ecl.2011.02.002 PMC3094542

[25] Bravo EL, Tagle R. Pheochromocytoma: state-of-the-art and future prospects. Endocr Rev. 2003;24(4):539‐553. 12920154 10.1210/er.2002-0013

[26] Petrák O, Krátká Z, Holaj R, et al Cardiovascular complications in pheochromocytoma and paraganglioma: does phenotype matter? Hypertens. 2024;81(3):595‐603. 10.1161/HYPERTENSIONAHA.123.21902 38152977

[27] Lawes CM, Vander Hoorn S, Rodgers A. Global burden of blood-pressure-related disease, 2001. Lancet. 2008;371(9623):1513‐1518. 18456100 10.1016/S0140-6736(08)60655-8

[28] Lloyd-Jones DM, Evans JC, Levy D. Hypertension in adults across the age spectrum: current outcomes and control in the community. JAMA. 2005;294(4):466‐472. 16046653 10.1001/jama.294.4.466

[29] Franklin SS, Larson MG, Khan SA, et al Does the relation of blood pressure to coronary heart disease risk change with aging? The framingham heart study. Circulation. 2001;103(9):1245‐1249. 11238268 10.1161/01.cir.103.9.1245

[30] Araujo-Castro M, Centeno RG, López-García MC, et al Risk factors for intraoperative complications in pheochromocytomas. Endocr Relat Cancer. 2021;28(11):695‐703. 34379605 10.1530/ERC-21-0230

[31] Shao Y, Chen R, Shen ZJ, et al Preoperative alpha blockade for normotensive pheochromocytoma: is it necessary? J Hypertens. 2011;29(12):2429‐2432. 22025238 10.1097/HJH.0b013e32834d24d9

[32] Fu SQ, Wang SY, Chen Q, Liu YT, Li ZL, Sun T. Laparoscopic versus open surgery for pheochromocytoma: a meta-analysis. BMC Surg. 2020;20(1):167. 32711496 10.1186/s12893-020-00824-6 PMC7382066

[33] Parente A, Verhoeff K, Wang Y, et al Robotic and laparoscopic adrenalectomy for pheochromocytoma: an international multicenter study. Eur Urol Focus. 2024:S2405-4569(24)00168-8. Doi: 10.1016/j.euf.2024.09.001 39278764

[34] Schimmack S, Kaiser J, Probst P, Kalkum E, Diener MK, Strobel O. Meta-analysis of α-blockade versus no blockade before adrenalectomy for phaeochromocytoma. Br J Surg. 2020;107(2):e102‐e108. 31903584 10.1002/bjs.11348

[35] Groeben H, Walz MK, Nottebaum BJ, et al International multicentre review of perioperative management and outcome for catecholamine-producing tumours. Br J Surg. 2020;107(2):e170‐e178. 31903598 10.1002/bjs.11378 PMC8046358

